# Age-related GABAergic differences in the primary sensorimotor cortex: A multimodal approach combining PET, MRS and TMS

**DOI:** 10.1016/j.neuroimage.2020.117536

**Published:** 2020-11-10

**Authors:** Koen Cuypers, Melina Hehl, June van Aalst, Sima Chalavi, Mark Mikkelsen, Koen Van Laere, Patrick Dupont, Dante Mantini, Stephan P. Swinnen

**Affiliations:** a Department of Movement Sciences, Movement Control & Neuroplasticity Research Group, Group Biomedical Sciences, KU Leuven, Heverlee, Belgium; b REVAL Research Institute, Faculty of Rehabilitation Sciences, Hasselt University, Agoralaan Building A, 3590 Diepenbeek, Belgium; c Nuclear Medicine and Molecular Imaging, Department of Imaging and Pathology, KU Leuven, and University Hospitals UZ Leuven, Leuven, Belgium; d Russell H. Morgan Department of Radiology and Radiological Science, The Johns Hopkins University School of Medicine, Baltimore, MD, USA; e F.M. Kirby Research Center for Functional Brain Imaging, Kennedy Krieger Institute, Baltimore, MD, USA; f Laboratory for Cognitive Neurology, Department of Neurosciences, KU Leuven, Leuven, Belgium; g Brain Imaging and Neural Dynamics Research Group, IRCCS San Camillo Hospital, Venice, Italy; h KU Leuven, Leuven Brain Institute (LBI), Leuven, Belgium

**Keywords:** Aging, GABA, PET, MRS, TMS

## Abstract

Healthy aging is associated with mechanistic changes in gamma-aminobutyric acid (GABA), the most abundant inhibitory neurotransmitter in the human brain. While previous work mainly focused on magnetic resonance spectroscopy (MRS)-based GABA+ levels and transcranial magnetic stimulation (TMS)-based GABA_A_ receptor (GABA_A_R) activity in the primary sensorimotor (SM1) cortex, the aim of the current study was to identify age-related differences in positron emission tomography (PET)-based GABA_A_R availability and its relationship with GABA+ levels (i.e. GABA with the contribution of macromolecules) and GABA_A_R activity. For this purpose, fifteen young (aged 20–28 years) and fifteen older (aged 65–80 years) participants were recruited. PET and MRS images were acquired using simultaneous time-of-flight PET/MR to evaluate age-related differences in GABA_A_R availability (distribution volume ratio with pons as reference region) and GABA+ levels. TMS was applied to identify age-related differences in GABA_A_R activity by measuring short-interval intracortical inhibition (SICI). Whereas GABA_A_R availability was significantly higher in the SM cortex of older as compared to young adults (18.5%), there were neither age-related differences in GABA+ levels nor SICI. A correlation analysis revealed no significant associations between GABA_A_R availability, GABA_A_R activity and GABA+ levels. Although the exact mechanisms need to be further elucidated, it is possible that a higher GABA_A_R availability in older adults is a compensatory mechanism to ensure optimal inhibitory functionality during the aging process.

## 1. Introduction

Gamma-aminobutyric acid (GABA), the main inhibitory neurotransmitter in the human brain, plays a crucial role in human motor behavior (Boy et al., 2010; Long et al., 2014; Paredes and Agmo, 1992; Schmidt-Wilcke et al., 2018). However, aging results in alterations of the GABAergic system and these have been linked with deficits in inhibitory control (Cuypers et al., 2018; Hermans et al., 2018a; Pauwels et al., 2018 2019; Heise et al., 2013; Levin et al., 2014), such as increased reaction times (Bedard et al., 2002; Hermans et al., 2019; Jordan and Rabbitt, 1977), impaired motor coordination (Heuninckx et al., 2004; Serrien et al., 2000; Swinnen, 1998) and reduced sensorimotor function (Calautti et al., 2001; Hehl et al., 2020). Along the same line, age-related impairments in sensorimotor performance are associated with less segregated sensorimotor brain networks and reduced GABA levels in the primary sensorimotor (SM1) cortex (Cassady et al., 2019). There is converging evidence that GABA levels decrease with advancing age in various regions of the brain (Hermans et al., 2018a; Gao et al., 2013; Grachev and Apkarian, 2001; Porges et al., 2017), including SM1 (Cassady et al., 2019; Chalavi et al., 2018; Grachev et al., 2001; Cuypers et al., 2020). Whereas local GABA levels can be accurately quantified using magnetic resonance spectroscopy (MRS), the amount of GABA receptor activity can be assessed by transcranial magnetic stimulation (TMS) (Cuypers and Marsman, 2020). Depending on the protocol, activity of the fast ionotropic GABA_A_ receptors (GABA_A_R) or slower acting metabotropic GABA_B_R can be measured with TMS (Ilic et al., 2002; McDonnell et al., 2006; Werhahn et al., 1999; Ziemann et al., 1996a).

In the current aging study, the focus is on GABA_A_Rs, the predominant type of GABA receptors in the brain (Bowery et al., 1987), which is estimated to be available in 20–50% of all synapses (Nutt and Malizia, 2001). Previous TMS studies investigating age-related differences in GABA_A_R activity in the primary motor cortex (M1) have revealed mixed results. Although there seems to be stronger evidence for an age-related decrease of GABA_A_ergic inhibition (Heise et al., 2013; Hermans et al., 2018b; Peinemann et al., 2001; Marneweck et al., 2011), some studies reported no differences (Hehl et al., 2020; Stevens-Lapsley et al., 2013; Wassermann, 2002) or even an increased inhibition (Kossev et al., 2002; McGinley et al., 2010). In addition, no significant associations between TMS-based GABA_A_R activity and MRS-based GABA levels in SM1 have been reported so far (Cuypers et al., 2020; Hermans et al., 2018b; Dyke et al., 2017; Mooney et al., 2017; Tremblay et al., 2013), suggesting that these two types of measurement represent different properties of the GABAergic system. While TMS reflects an indirect measure of intracortical GABAergic inhibition (Di Lazzaro et al., 2006; Kujirai et al., 1993), MRS is suggested to provide a measure of extrasynaptic GABA tone (Stagg et al., 2011a, 2011b).

Assessment of GABA_A_R availability using positron emission tomography (PET) provides another surrogate metric of GABAergic inhibition. However, evidence regarding in-vivo derived GABA_A_R availability obtained using PET has been absent in the context of healthy aging. Only a recent study using iodine-123-iomazenil single photon emission computed tomography (IMZ SPECT) found an increase of GABA_A_R availability in the left prefrontal cortex (PFC) in older as compared to younger adults (Tobinaga et al., 2019). Based on these findings, it was suggested that an increase in postsynaptic GABA_A_R availability is likely a mechanism to compensate for the reduction in synaptic GABA levels in this region (Tobinaga et al., 2019; Caspary et al., 2008; Pomares et al., 2020). This suggestion is in accordance with evidence showing a significant negative association between GABA_A_R binding and plasma GABA levels (Klumpers et al., 2010). As compared to TMS- and MRS-based GABAergic measures, PET with [^11^C]flumazenil, a radiotracer that binds to the benzodiazepine site of GABA_A_Rs, allows a more direct quantification of GABA_A_ inhibitory mechanisms at the level of the synapse (Kujala et al., 2015; Finnema et al., 2015) and might be a superior surrogate for the study of GABAergic inhibition as compared to GABA levels or measures of GABA_A_R activity. However, no studies have yet explored the associations between a PET-based measure of GABA_A_R availability and a MRS-based measure of GABA levels, and/or a TMS-based measure of GABA_A_R activity. Recently, our group already studied GABA+ levels (Hermans et al., 2018a; Chalavi et al., 2018) and GABA_A_R activity (Hermans et al., 2019) and their relationship (Cuypers et al., 2020; Hermans et al., 2018b) in the context of healthy aging. However, this is the first time that we associate these metrics to PET-based GABA_A_R availability.

Here, our aims were twofold. The first aim was to identify whether and how GABA_A_R availability in SM1 differs between young and older adults. Secondly, we investigated the relationships between PET-based GABA_A_R availability, MRS-based GABA+ levels and TMS-based GABA_A_R activity. We focused on the SM1 region because it plays a key role in sensorimotor function with advancing age (Cassady et al., 2019; He et al., 2016). To the best of our knowledge, this is the first study to investigate age-related GABAergic differences in SM1 using a multimodal approach combining PET, MRS and TMS. Firstly, we hypothesized to observe an increased GABA_A_R availability in SM1 of older adults (Tobinaga et al., 2019). Secondly, we expected to find an age-related decrease in GABA+ levels (Cassady et al., 2019; Chalavi et al., 2018; Grachev et al., 2001) and GABA_A_R activity (Heise et al., 2013; Hermans et al., 2018b; Peinemann et al., 2001; Marneweck et al., 2011) in SM1. And finally, based on (Tobinaga et al., 2019), we tentatively hypothesized that a decrease in GABA+ levels will covary with an age-related increase in GABA_A_R availability.

## 2. Methods

### 2.1. Participants

Fifteen young [aged 20–28 years, 23.4 ± 2.2 (mean ± SD); 7 males] and fifteen older [aged 65–80 years, 70.7 ± 4.1 (mean ± SD); 7 males] participants were included in this study (see Table 1 for detailed participant characteristics). All participants were right-handed according to the Edinburg Handedness Inventory (Oldfield, 1971) [lateralization quotient (mean ± SD), young adults: 91.4 ± 9.7; older adults: 95.6 ± 7.9] and had normal or corrected-to-normal vision. At the start of the study, participants also completed the Mini Mental State Examination (Folstein et al., 1975; Molloy et al., 1991) [MMSE (mean ± SD), young adults: 29.8 ± 0.4, range 29–30; older adults: 29.2 ± 1.0, range 27–30; overall scores can range from 0 to 30; the cut point for normal cognitive function is often set at 24 (Creavin et al., 2016)], and the Baecke Questionnaire of Habitual Physical Activity (self-reported) (Baecke et al., 1982; Voorrips et al., 1991) (mean ± SD, young adults: 8.1 ± 2.0; older adults: 8.3 ± 1.4; overall scores can range from 3 – least physically active, to 15 – most physically active). None of the participants reported medication intake affecting the central nervous system over the last month or a history of neurological, psychiatric, cardiovascular, or neuromuscular disorders. Participants were screened for magnetic resonance imaging (MRI) (Dill, 2008) and TMS contraindications (Wassermann, 2002) and provided written informed consent prior to the start of the experiment. The protocol was approved by the local Ethics Committee Research of UZ/KU Leuven (study number: S60542) and was conducted in accordance with the latest version of the Declaration of Helsinki.

### 2.2. Experimental design

This cross-sectional study consisted of two experimental sessions. In the first session, high-resolution anatomical MRI, MRS and PET data were collected using a hybrid PET/MR system at the University Hospital Leuven. MRS data were collected during the 60-min PET scan at approximately 20 min after the onset of the PET scan. In the second session, TMS was applied to assess resting-state short-interval intracortical inhibiton (SICI) over the left M1. The timing between the TMS and the imaging session was 22.1 ± 20.2 days (mean ± SD). Participants participated first in the imaging session and subsequently in the TMS session, with exception of two participants whose imaging session was replanned to a later timing due to suboptimal tracer production at the initial session. Please note that throughout this work, the terminology M1 refers strictly to the region targeted with TMS. In turn, MRS and PET data were collected from a broader volume of interest (VOI) which was centered over the left hand knob (Yousry et al., 1997), and is referred to as SM1. The VOI was placed with the goal to maximize the amount of gray matter (GM) relative to white matter (WM) and cerebrospinal fluid (CSF), while taking into account each individual participant’s anatomy.

### 2.3. Hybrid PET/MR imaging

#### 2.3.1. [^11^C]Flumanzenil PET

Each participant was injected with an average bolus of 301 ± 39 MBq (mean ± SD) of [^11^C]flumazenil (see supplementary document of Van Laere et al. (2008) for details regarding the radiosynthesis) through an intravenous catheter at the start of the PET scan. Dynamic PET images were acquired for 60 min post-injection in listmode on the 3T GE Signa hybrid time-of-flight (TOF) PET/MR system (General Electric Healthcare, Milwaukee, MI, USA).

#### 2.3.2. MRI

**Fig. 1.**
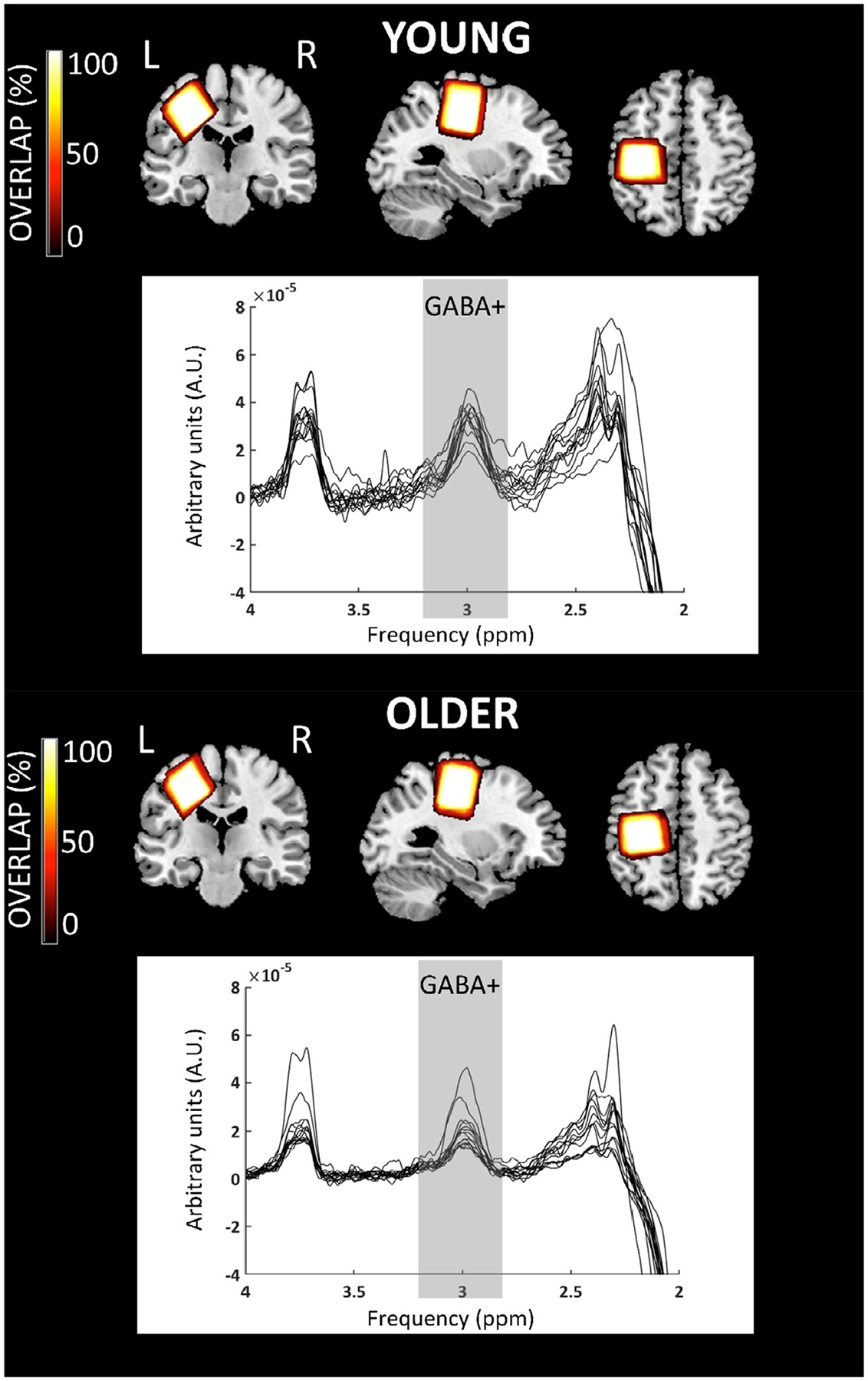
Overview of the magnetic resonance spectroscopy (MRS) [and similar for positron emission tomography (PET)] voxel positions and raw spectra for older and young adults. The color bar indicates the overlap of the individual voxels, with bright color indicating a high overlap and dark color a low overlap. The GABA+ peak is expected at 3.0 parts per million (ppm) of the proton frequency and is highlighted in gray.

A 3D Brain Volume (BRAVO) high-resolution T1-weighted anatomical image (repetition time = 8.4 ms; echo time = 3.2 ms; 1 × 1 × 1 mm^3^ voxels; field of view = 256 × 256 mm^2^; 166 sagittal slices; flip angle = 12°) and MRS spectra were acquired. MRS was performed to acquire GABA+ levels. More specific, the MEGA-PRESS [14 msec editing pulses at 1.9 parts per million of the proton frequency (ppm) and 7.46 ppm; repetition time = 2000 ms; echo time = 68 ms; 160 on and 160 off averages; 4096 points; 2 kHz spectral width; CHESS water suppression (Haase et al., 1985); scan duration: 11 min 28 s] was used to measure GABA levels with the contribution of macromolecules commonly referred to as GABA+ (Mullins et al., 2014). For each participant, the SM1 voxel (3 × 3 × 3 cm^3^) was centered over the left hand knob (Yousry et al., 1997), parallel to the anterior-posterior axis. One surface was parallel to the cortical surface in the coronal and axial views (see Fig. 1).

### 2.4. Image processing

#### 2.4.1. [^11^C]Flumanzenil PET

Data were reconstructed using vendor specific software (software version MP24.0R03) into 22 frames (4 × 15 s, 4 × 30 s, 4 × 1 min, 4 × 2 min, 3 × 5 min and 3 × 10 min). An MR-based attenuation correction using the zero-echo time (ZTE) sequence was used to correct for attenuation (Schramm et al., 2019). All frames were reconstructed using TOF ordered-subset expectation maximization (TOF OSEM) with 6 iterations, 28 subsets and isotropic Gaussian post-smoothing with a full-width-at-half-maximum (FWHM) of 4.5 mm and a voxel size of 1.56 × 1.56 × 2.78 mm^3^. All data were corrected for deadtime, randoms and scatter.

Further processing of the PET images was performed offline using a combination of the statistical parameter mapping (SPM 12) toolbox and in-house customized MATLAB scripts (R2018a, The MathWorks Inc., Natick, MA, USA). First, frames were realigned to correct for motion, using an average image of the first 5 min (frames 1–9) as a reference. Second, PET images were rigidly matched to the participant’s 3D T1-weighted MR image using the Normalized Mutual Information criterion. Third, the MR images were segmented into GM, WM and CSF. Fourth, PET and MR images were normalized to Montreal Neurological Institute (MNI) space using the deformation field obtained during the segmentation process. A customized pons VOI served as the reference region (Salmi et al., 2008). It consisted of an ellipse of 8 mm × 4 mm that was positioned on three consecutive planes (Klumpers et al., 2008), resulting in a cylindric pons VOI. Finally, Ichise’s Multilinear Reference Tissue Model 2 (MRTM2) was used to calculate the distribution volume ratio (DVR), which is a linear function of receptor availability, using the pons as reference region. The parameter k_2_’ which is used in the MRTM2 model was determined based on the simplified reference tissue model applied on the time-activity curve in a receptor rich region (defined based on the GM segmentation > 0.3). PET images were corrected for partial volume effects using the region-based voxelwise (RBV) method (Thomas et al., 2011) based on the geometric transfer matrix (GTM) method (Rousset et al., 1998) and the tissue class information. This correction was performed because athophied GM regions (which are expected in older adults) suffer more from partial volume effects (Thomas et al., 2011).

In this work only the PET data from the left SM1 VOI were analyzed. Moreover, PET data on the spatially normalized maps were extracted from the SM1 VOI being identical to the MRS SM1 VOI to perform the VOI analysis between the young and older group (see Fig. 1 for an overview of the average VOI location, identical for PET and MRS). For each participant, the GM masked median DVR value in the VOI was calculated. Here, median values were chosen over average values because the median is robust for extreme high or low values which might result from over- or undercorrection for partial volume effects using the RBV method.

#### 2.4.2. MRS

MRS data were processed offline using the Gannet 3.0 toolbox (http://www.gabamrs.com/downloads) (Edden et al., 2014). Individual frequency domain spectra were frequency- and phase-corrected using spectral registration and filtered with a 3 Hz exponential line broadening. The area under the edited GABA+ signal at 3 ppm was estimated (see Fig. 1 for the raw spectra). This editing scheme coedits approximately 50% macromolecules at 3 ppm, which are coupled to spins at 1.7 ppm, also inverted by editing pulses. GABA+ and unsuppressed water signals were modeled using a single Gaussian peak with a five parameter Gaussian model and a Gaussian-Lorentzian model, respectively (Edden et al., 2014). Next, MRS voxels were co-registered to the T1-weighted image and segmented to determine fractions of the different tissue types (GM, WM and CSF). Based on these tissue fraction measurements, tissue-corrected GABA+ values were obtained for each voxel (Edden et al., 2014). Tissue correction is necessary as it is assumed that GABA+ levels are negligible in CSF and twice as high in GM as compared to WM (Edden et al., 2014). Additionally, tissue-specific relaxation and water visibility values were taken into account. Finally, GABA+ levels were normalized to the average voxel composition of each age group (see Harris et al., 2015a, Eq. (6). This full tissue normalization results in a GABA+ value, which takes into account the average voxel tissue composition for the cohort. In addition, water frequency drift and fit errors of the GABA+ peak were calculated to provide a measure of MRS data quality (see Table 2). Note that GABA+ levels were reported in institutional units (I.U.). More specifically, they were quantified from the ratio of the integral of the edited GABA+ signal to the integral of the unsuppressed water signal multiplied by a scaling factor to account for tissue-specific differences in T1 and T2 relaxation times of GABA+ and water and the editing efficiency (Mullins et al., 2014; Harris et al., 2015b).

### 2.5. Transcranial magnetic stimulation (TMS)

TMS was performed using a figure-of-eight coil with an inner wing diameter of 70 mm connected to a Magstim BiStim^2^ (Magstim, Whitland, Dyfed, UK) and combined with electromyographic (EMG) measurements to assess changes in motor evoked potentials (MEPs). Prior to experimental measurements, single-pulse TMS was used to determine the optimal stimulation location (hotspot) of the left M1. For this purpose, each participant’s head was covered with a cap, labeled with an orthogonal 1 × 1 cm^2^ coordinate system, with references to anatomical landmarks (nasion, inion, and left and right auditory meatus). TMS was applied to the scalp with the coil handle rotated 45° away from the midsagittal line (Brasil-Neto et al., 1992). The hotspot was defined as the scalp location resulting in the highest MEP in the relaxed first dorsal interosseous (FDI) muscle averaged over five consecutive stimuli. The coil position and orientation at the hotspot were co-registered to the individual anatomical MR image using an MRI-based neuronavigation system (VISOR 2, ANT Neuro, the Netherlands). The resting motor threshold (rMT) was defined as the lowest stimulation intensity evoking MEPs with an amplitude larger than 50 μV peak-to-peak in at least five of ten consecutive trials at rest (Rossini et al., 1999). GABA_A_R activity was indirectly assessed using a paired-pulse SICI protocol. Specifically, a conditioning stimulus (CS) was followed by a test stimulus (TS) with an interstimulus interval of 3 ms. The CS was set at 80% rMT (Hermans et al., 2018b; Ziemann et al., 1996b) and the TS was adjusted to elicit unconditioned MEP amplitudes of approximately 1 mV peak-to-peak (Heise et al., 2013; Hermans et al., 2018b). In total 15 paired (CS + TS) and 15 single (TS alone) pulses were administered and MEPs were averaged per condition. SICI was expressed as: (1 − (MEP_paired-pulse_/MEP_single-pulse_)) * 100. A higher positive value implies more inhibition, while higher negative values indicate higher disinhibition.

EMG signals from the right FDI muscle were continuously recorded (Bagnoli-16, Delsys Inc, Boston, USA). After amplification (gain = 1000), bandpass filtering (4–1500 Hz) and 50/60 Hz noise elimination (Humbug, Quest Scientific, North Vancouver, Canada), the recorded EMG signals were digitized at 5000 Hz (CED Signal Version 6.0, Cambridge Electronic Design, Cambridge, UK) and stored on a computer for offline analysis.

### 2.6. Statistics

Prior to analysis, all data were screened for outliers. For GABA_A_R availability and GABA+ levels, data were qualified as an outlier and excluded from the analyses if values exceeded the group mean with more than 3 standard deviations (SD). For PET, all data points were included in the analysis. For MRS, one young adult was excluded from the analysis due to lipid contamination, after visual inspection of the spectrum. Individual datasets were also inspected for frequency drift and fit errors. Spectra with a fit error below 12% are generally considered to be of sufficient quality (Edden et al., 2014). For TMS, individual MEPs were excluded from analysis (10 of 900 trials were excluded; ~1%) if the root mean square EMG exceeded 20 *μ*V.

JMP Pro 14 (SAS Institute Inc, Cary, NC, USA) was used for statistical analysis. If assumptions for normality of the data were fulfilled (Shapiro–Wilk W-Test) parametric tests were performed, otherwise a non-parametric statistical test was applied. The effect size was calculated using Cohen’s *d* (*d*_Cohen_). The significance level was set to *α* = 0.05. A Bonferroni correction was applied to correct for multiple comparisons.

## 3. Results

### 3.1. PET

**Fig. 2.**
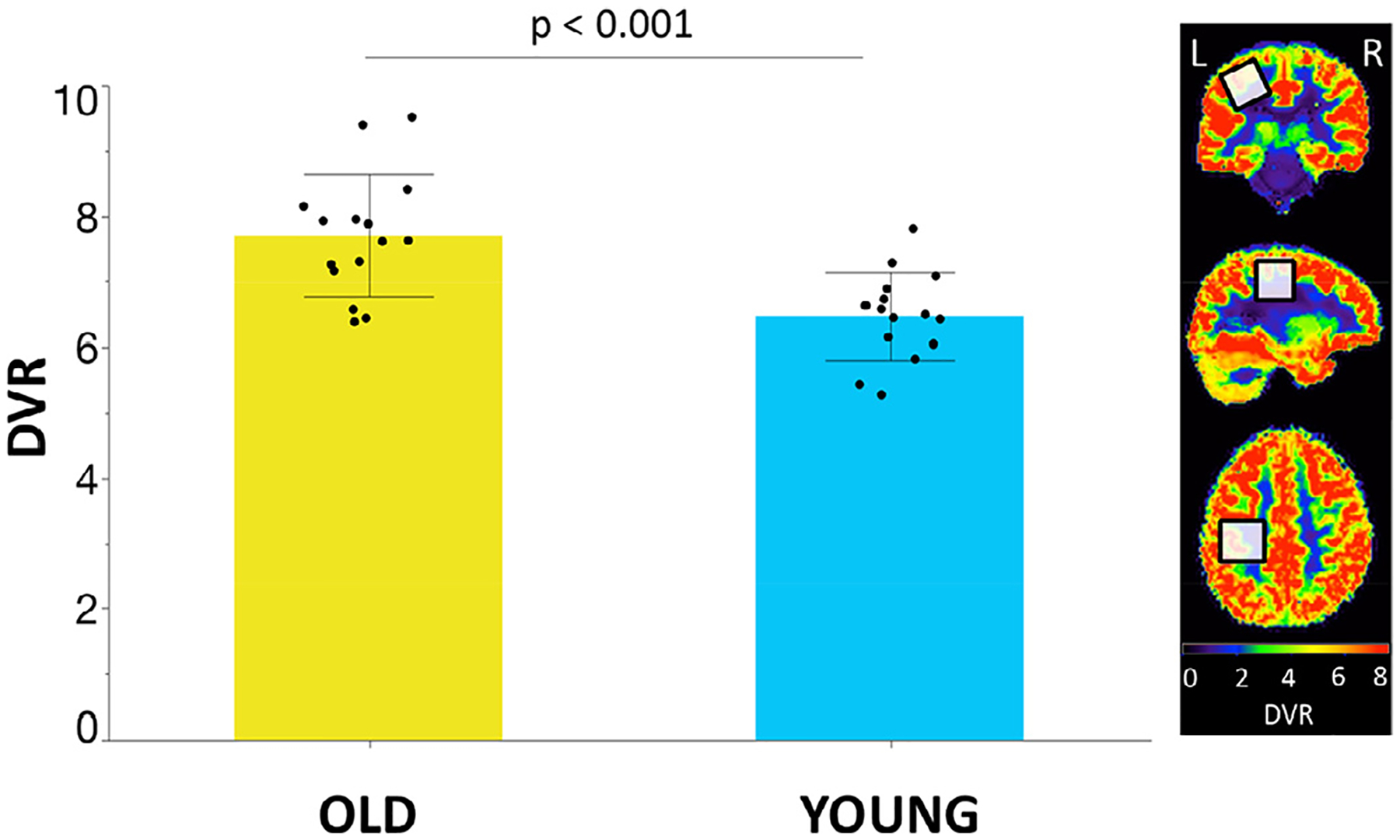
Gamma-aminobutyric acid type A (GABA_A_R) availability for the left primary sensorimotor (SM1) voxel expressed in distribution volume ratio (DVR) values of [^11^C]flumazenil in older (yellow bar) and young (blue bar) adults. Black dots represent the individual median DVR values. Bar plots refer to the median values; error bars represent the standard deviation.

[^11^C]-Flumazenil DVR values in SM1 differed significantly between young and older adults, showing a higher GABA_A_R availability in older as compared to young adults (difference = 18.5%; DVR: mean ± SD, young adults: 6.5 ± 0.7; older adults: 7.7 ± 0.9; effect size d_Cohen_ = 1.5; independent samples *t*-test; *t*(28) = −4.1, *p* < 0.001; see Fig. 2).

### 3.2. MRS

**Fig. 3.**
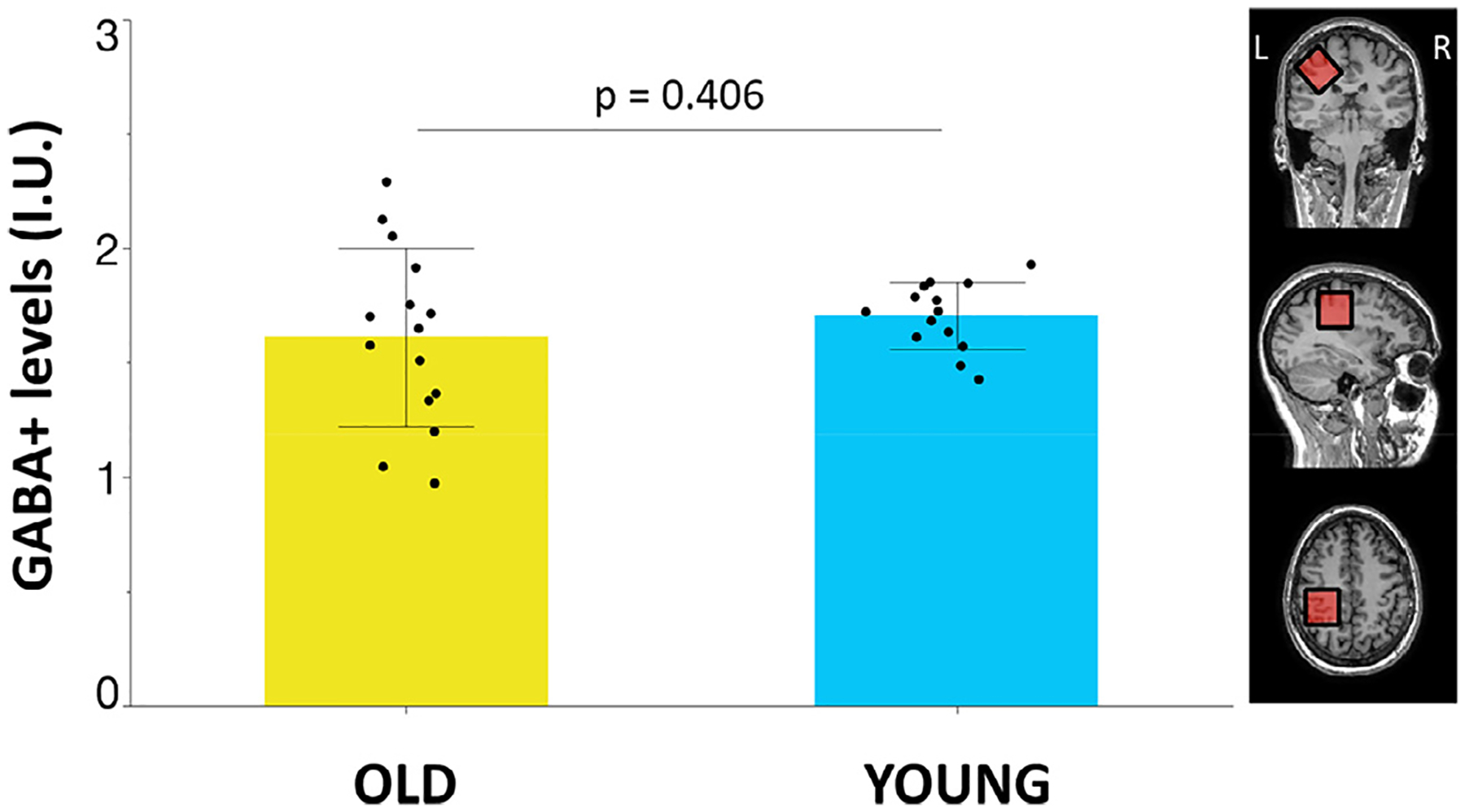
Tissue-corrected gamma-aminobutyric acid with the contribution of macromolecules (GABA+) levels [institutional units (I.U.)] in the left primary sensorimotor (SM1) voxel in older (yellow bar) and young (blue bar) adults. Black dots represent the individual GABA+ levels. Bar plots refer to the mean values; error bars represent the standard deviation.

Mean GABA+ levels did not differ between groups [GABA+ levels (I.U.): mean ± SD, young adults: 1.70 ± 0.15; older adults: 1.61 ± 0.39; effect size *d*_Cohen_ = 0.3; independent samples *t*-test; t(27) = 0.8, *p* = 0.406; see Fig. 3]. Tissue fractions and quality metrics of the MRS data are presented in Table 2.

### 3.3. TMS

**Fig. 4.**
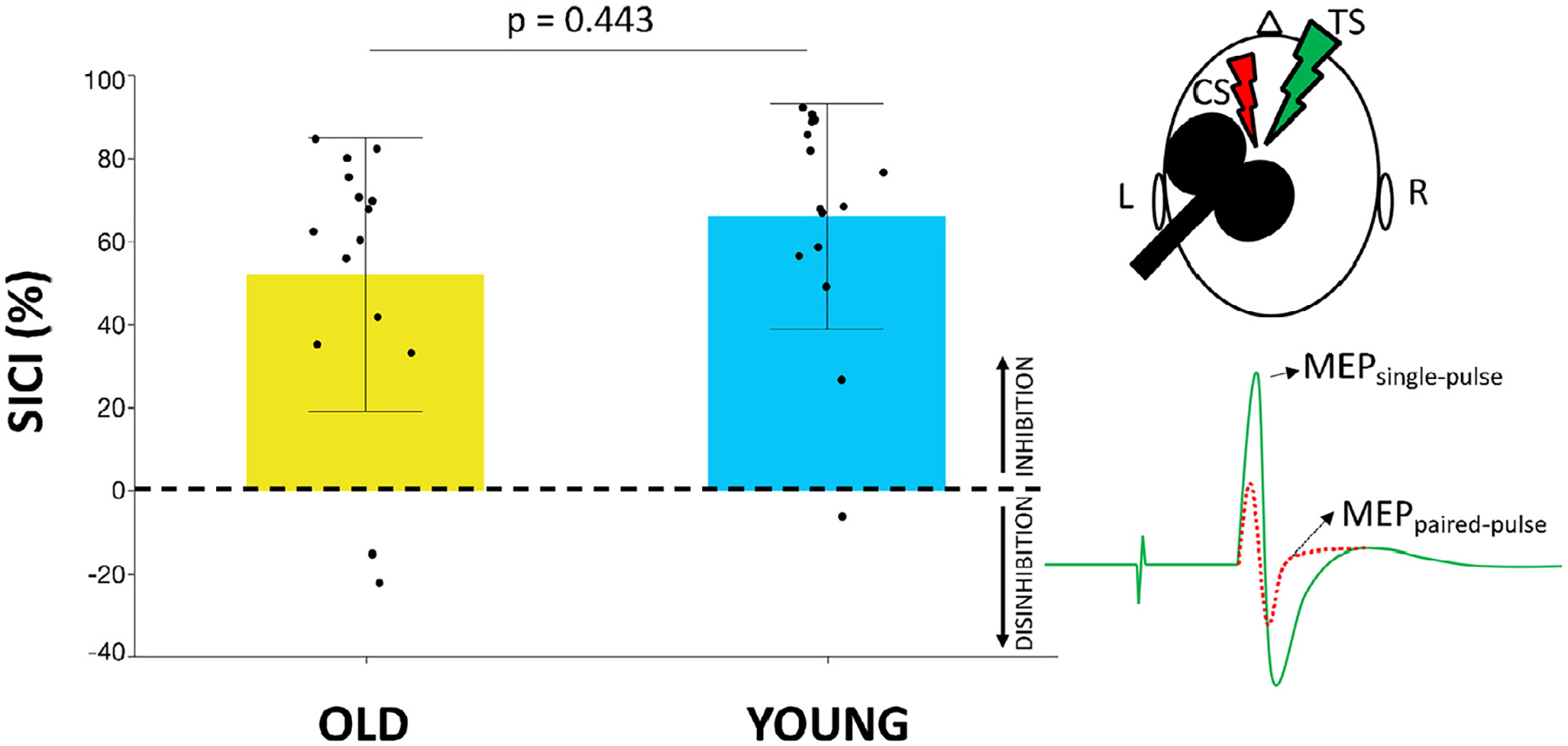
short-interval intracortical inhibition (SICI) in the left primary motor cortex (M1) in older (yellow bar) and young (blue bar) adults. Black dots represent individual SICI values. Bar plots refer to the mean values; error bars represent the standard deviation. SICI was defined as: (1 − [Motor evoked potential (MEP)_paired-pulse_/MEP_single-pulse_]) * 100. Higher positive values indicate higher inhibition, while higher negative values indicate higher disinhibition. The right part of the figure illustrates the SICI principle. MEP_single-pulse_ is assessed after administering a single test stimulus (TS) (visualized as the green ‘lightning’). MEP_paired-pulse_ is obtained when the TS is preceded by a conditioning stimulus (CS) (visualized as the red ‘lightning’).

Although for both groups, the MEPs following paired pulses were significantly suppressed as compared to MEPs induced by single pulses (paired *t*-test; both, *p* < 0.001), the mean SICI did not differ significantly between groups, indicating a similar level of GABA_A_R activity (SICI: mean ± SD, young adults: 66.2% ± 27.2; older adults: 52.1% ± 33.0; effect size *d*_Cohen_ = 0.5; Kruskal–Wallis Test; *Z* = 0.8, *p* = 0.443; see Fig. 4).

### 3.4. Associations between PET, MRS and TMS

**Fig. 5.**
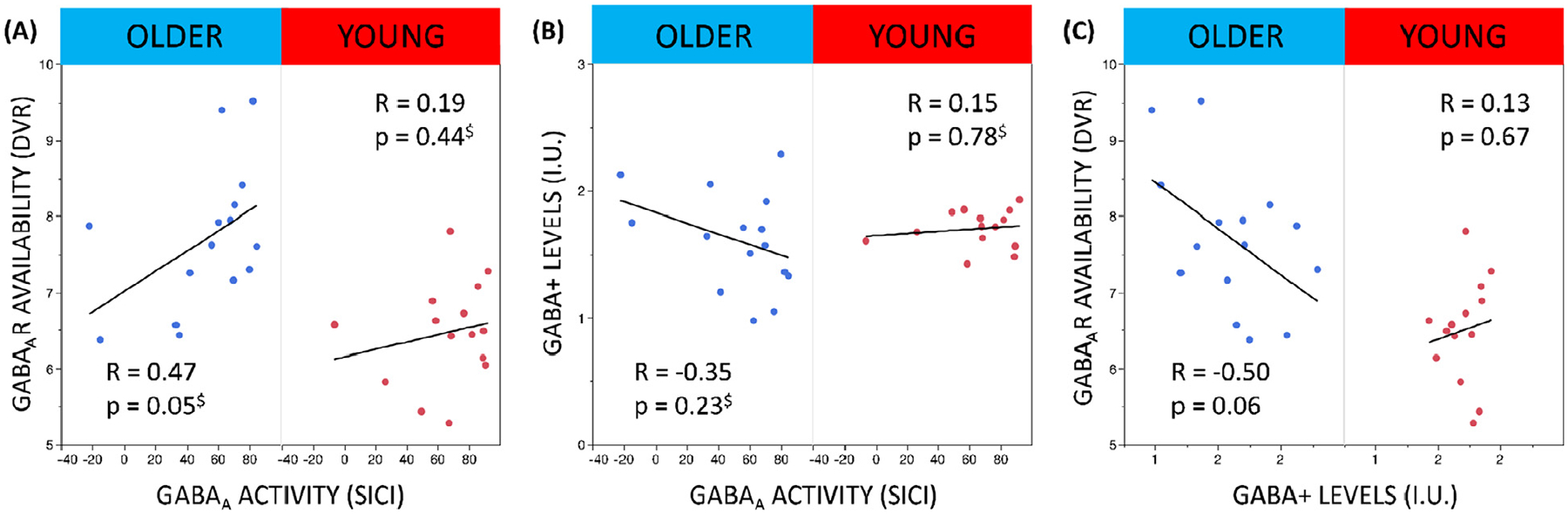
Illustration of the associations between positron emission tomography (PET)-based gamma-aminobutyric acid type A (GABA_A_R) availability in left primary sensorimotor (SM1) voxel, magnetic resonance spectroscopy (MRS)-based GABA with the contribution of macromolecules (GABA+) levels [institutional units (I.U.)] in the left SM1 voxel and TMS-based GABA_A_R activity in the left primary motor cortex (M1) in young and older adults. The critical p-value is 0.025 (based on the Bonferroni correction for multiple comparisons). ^$^ indicates that a Spearman correlation test was used instead of a Pearson correlation test.

No significant associations between PET, MRS and TMS were identified (all, *p* > 0.05; see Fig. 5a–c).

## 4. Discussion

The present study yielded three major findings. First, GABA_A_R availability in SM1 was higher in older as compared to young adults. Second, GABA+ levels and GABA_A_R activity were not significantly different between age groups. Finally, no significant associations were observed between GABA+ levels, GABA_A_R availability and GABA_A_R activity.

Firstly, we observed an age-related increase in GABA_A_R availability in SM1, which was in line with our hypothesis. So far, only one study has explored GABA_A_R availability in relation to aging, using IMZ SPECT (Tobinaga et al., 2019), a technique with a lower spatial resolution as compared to time-of-flight PET imaging employed in the current study (Rahmim and Zaidi, 2008). However, this IMZ SPECT study focused on age-related differences (age range from 22 to 59 years) in the left PFC, rather than in the left SM1 region, and demonstrated significantly more relative GABA_A_R availability in the left PFC with advancing age, which was assumed to reflect a compensatory mechanism for the overall reduction in GABA+ levels. Alternative explanations for our current findings can be inferred from animal research indicating that age-related differences in GABA_A_R availability are possibly a result of altered GABA_A_R subunit composition, which in turn may alter affinity of [^11^C]flumazenil radioligand binding (Rissman et al., 2007; Rissman and Mobley, 2011). GABA_A_Rs are mainly pentameric proteins, with the major isoform consisting of two *α*_1_, two *β*_2_ and one *γ*_2_ subunits (see review Sigel and Steinmann, 2012). It has been established that instead of binding at the GABA site, flumazenil binds at the benzodiazepine site (between the *α* and *γ* subunits) of the GABA_A_R subtypes that contain *α*_1_, *α*_2_, *α*_3_ or *α*_5_ subunits, while GABA_A_R subtypes containing *α*_4_ or *α*_6_ subunits are benzodiazepine- and consequently flumazenil-insensitive (Sigel and Ernst, 2018). Furthermore, affinity of [^11^C]-flumazenil is higher to *α*_1_ subunits as compared to *α*_2_, *α*_3_ or *α*_5_ subunits (Van Laere et al., 2008; Ruano et al., 1995, 1991). Additionally, the temporal and regional diversity of age-related differences in subunit composition in humans is still unclear and dynamic changes (increases or decreases) in *α*, *β* and *γ* subunit expression are observed in human and non-human primates (Rissman and Mobley, 2011; Duncan et al., 2010; Fillman et al., 2010; Hashimoto et al., 2008). Therefore, it can be assumed that subunit composition alterations might be crucial to ascertain optimal inhibitory functionality during the aging process. Another possible explanation for the increased GABA_A_R availability is that this is a compensatory strategy for an age-related decrease in GABA_B_R binding (Milbrandt et al., 1994 1996), that similarly to GABA_A_Rs mediate inhibitory action. However, it is not possible to verify this speculation as GABA_B_R cannot be quantified in vivo yet (Andersson et al., 2019). Moreover, an increase in GABA_A_R availability was observed in fibromyalgia patients (Pomares et al., 2020), and this finding was suggested to demonstrate that the GABA_A_Rs might even shift functionality from inhibitory to excitatory, a disease mechanism that has also been associated with epilepsy (Robel and Sontheimer, 2016). However, this explanation is rather unlikely in the context of healthy aging as the current study found a positive association between GABA_A_R availability and GABA_A_R activity in older adults (as discussed further below). As mentioned earlier, studies investigating age-related GABA_A_R availability in vivo using PET are scarce. A recent postmortem study reported that the GABAergic system was not characterized by age- and sex-specific differences of glutamic acid decarboxylase (GAD), GABA receptor subunits and GABA transporter (GAT) in sensory and motor regions of the human brain (Pandya et al., 2019). However, it should be noted that obtaining reliable results from postmortem human tissue samples is challenging due to various issues such as tissue collection, handling and storage as well as heterogeneity of brains (Rissman et al., 2007).

Secondly, we hypothesized to observe age-related decreases in GABA+ levels and GABA_A_R activity, with the observed increase in GABA_A_R availability as a possible compensatory strategy for this reduction. However, although several studies (Tobinaga et al., 2019; Caspary et al., 2008; Pomares et al., 2020) supportingly have suggested that age-related differences in GABA_A_R availability may reflect a postsynaptic compensation for age-related loss in presynaptic GABA release, our data does not support this assumption as we did not observe a significant difference in GABA+ levels between age groups. This result should be interpreted in view of current inconsistencies in the MRS literature which occasionally reports an absence of age-related differences (Hermans et al., 2018b; Maes et al., 2018) as well as age-related decrease in GABA+ levels in SM1 (Cassady et al., 2019; Chalavi et al., 2018; Grachev et al., 2001; Cuypers et al., 2020). Similarly, our TMS experiment identified no significant age group differences in GABA_A_R activity. Comparably to the MRS literature, TMS studies dealing with age-related differences in GABA_A_R activity in M1 have revealed mixed results, i.e., reporting an age-related decrease of inhibition (Heise et al., 2013; Hermans et al., 2018b; Peinemann et al., 2001; Marneweck et al., 2011), no differences (Stevens-Lapsley et al., 2013; Wassermann, 2002) or even an increased inhibition (Kossev et al., 2002; McGinley et al., 2010). It should be noted that the lack of significant age-related differences in GABA+ levels and GABA_A_R activity might be due to the relatively small sample size. Whereas a sample size between 10 and 20 is considered reasonable for PET studies (Andreasen et al., 1996), higher sample sizes might be desirable for MRS and TMS experiments in order to achieve higher statistical power for detecting (subtle) differences between age groups. Alternatively, it is likely that GABA_A_R availability provides a more direct measure of GABAergic inhibition than GABA+ levels or GABA_A_R activity, which can lead to better detection of age-related differences as this measure is less influenced by factors that can contaminate the measurement of GABAergic inhibition. For instance, in the MRS technique, the resonance frequency of GABA is close to the frequencies of other metabolites, which is why GABA is often quantified (contaminated) together with macromolecules as GABA+ and signals from large voxels need to be acquired to obtain a reliable measure (Mullins et al., 2014). In addition, the in vivo concentration of GABA (1–2 mM) is at the lower end of the detectable range for MRS (Pomares et al., 2020) and MRS cannot be used to distinguish between neurotransmitter levels from the synaptic and the extracellular pool (Stagg et al., 2011a). Similarly, TMS-related MEPs are highly variable within and across participants (Biabani et al., 2018) due to physiological and technical factors such as background neural activity, environmental noise and coil positioning.

Finally, at the inter-individual level, our study revealed no significant associations between GABA+ levels, GABA_A_R availability and GABA_A_R activity. Our result revealed no significant association between GABA_A_R availability and GABA_A_R activity neither in older, nor in young adults. However, it is possible that an absence of a relationship, particularly in older adults (see Fig. 5a), might be explained by the low sample size. Moreover, with respect to GABA_A_R activity, which was measured using SICI, a pharmacological study suggested that SICI is mediated by the *α*_2_ and/or *α*_3_, and not by *α*_1_, subunits of the GABA_A_Rs (Di Lazzaro et al., 2006). In contrast, flumazenil, used to quantify GABA_A_R availability, binds at the benzodiazepine site of the GABA_A_Rs not only between the *α*_2_ or *α*_3_ and *γ* subunits, but also between subtypes of GABA_A_Rs bearing *α*_1_ or *α*_5_ and *γ* subunits, with likely a higher affinity to *α*_1_ subunits as compared to *α*_2_, *α*_3_ or *α*_5_ subunits (Ruano et al., 1995; Ruano et al., 1991). Moreover, the predominant subtype of GABA_A_Rs is composed of *α*_1_, *β*_2_ and *γ*_2_ subunits (McKernan and Whiting, 1996). These findings might suggest that the composition of GABA_A_Rs in the left SM1 is altered with advancing age. More specifically, it might be possible that a relationship between GABA_A_R activity and GABA_A_R availability emerges with advancing age due to altered ratios between the predominant subtype of GABA_A_Rs (containing the *α*_1_ subunit) and collateral subtypes of GABA_A_Rs (bearing *α*_2_ and *α*_3_ subunits). However, further research is needed to clarify this hypothesis. Apart from that, no significant associations were found between the other GABA metrics. For the association between GABA+ levels and GABA_A_R activity, there is substantial evidence for an absent relationship (Cuypers et al., 2020; Hermans et al., 2018b; Dyke et al., 2017; Mooney et al., 2017; Tremblay et al., 2013; Stagg et al., 2011b). As assumed by Stagg et al. (2011), GABA+ levels might be more closely linked to tonic inhibition, which originates from extracellular GABA acting on extrasynaptic GABA_A_Rs, than to vesicular GABA acting on synaptic GABA_A_Rs (Stagg et al., 2011 a 2011b). Furthermore, although we hypothesized that a decrease in GABA+ levels would covary with an age-related increase in GABA_A_R availability, only a non-significant trend was noticed for older but not for young adults (see Fig. 5c). Findings from preclinical studies evaluating the effect of mood stabilizers and antidepressants on GABAergic neurotransmission in mood disorders indicate complex interactions between GABA+ levels, GABA enzymes and GABA_A_R and GABA_B_R availability, which are dependent on the administered substrate (Brambilla et al., 2003). Keeping this in mind, it is challenging to directly relate GABA+ levels with GABA_A_R availability, without having access to other relevant GABAergic properties, for example GABA_B_R availability.

## 5. Limitations

There are some limitations in this study, which need to be addressed. First, only two age groups were investigated. Extending the age range or adding (middle) age groups might reveal a non-linear relationship between age and GABA_A_R availability. Although an increase in GABA_A_R availability may be indicative of a compensatory mechanism, it might be possible that GABA_A_R availability decreases again as people age further, as a consequence of brain deterioration and lack of compensatory strategies. Second, one should be aware of a possible selection bias and therefore be careful with generalizing our results to the general population. Moreover, it is possible that the older participants in the current study were ‘highly active’, being involved in various social and physical activities. Nevertheless, the young and older group self-reported comparable levels of physical activity (Baecke Questionnaire of Habitual Physical Activity). A third limitation is that the order of measurements of two participants differed from the other participants, due to tracer production issues at the initial session. However, re-analysis of the associations between GABA_A_R activity and respectively GABA_A_R availability and GABA+ levels, with these two participants excluded, did not change the main conclusion, stating that there are no significant associations between the different metrics. Fourth, the sample size in this study was relatively small. A higher sample size would have increased statistical power and generalizability of our results. A final limitation refers to the relatively large voxel size (3 × 3 × 3 cm^3^) used for GABA-edited MRS. Nonetheless, to ensure a sufficient signal-to-noise ratio and avoid long scanning times, a voxel of this size is commonly used for GABA-edited MRS and offers a realistic compromise between voxel size and signal quality (Mullins et al., 2014). Consequently, the VOI measured with MRS exceeds the region targeted with TMS. Therefore, GABA+ levels measured in this work are originating not only from M1, but also from the adjacent primary somatosensory cortex (S1).

## 6. Conclusion

We demonstrated that GABA_A_R availability in SM1 was higher in older as compared to young adults, while no significant differences were observed for GABA+ levels and GABA_A_R activity across age groups. Although a possible explanation for this increased GABA_A_R availability in older adults to date is a postsynaptic compensation for an age-related loss in presynaptic GABA release, our data does not support this assumption. Other mechanisms such as altered GABA_A_R subunit composition or a compensatory mechanism for an age-related decrease in GABA_B_R binding are also possible but cannot be assessed in vivo with the current techniques. Additionally, no significant associations between GABA+ levels, GABA_A_R availability and GABA_A_R activity were observed.

## Figures and Tables

**Table 1:** Participant characteristics. For each participant of each age group, gender (*M* = male, *F* = female), lateralization quotient (LQ), Mini Mental State Examimation score (MMSE) and Baecke score (Questionnaire of Habitual Physical Activity) are reported.

ID	AGE GROUP	GENDER	AGE	LQ	MMSE	BAECKE
1	OLDER	M	72	100.0	30	8.8
2	OLDER	M	65	100.0	30	9.5
3	OLDER	F	72	100.0	29	8.6
4	OLDER	F	68	100.0	29	6.4
5	OLDER	M	69	81.8	30	8.1
6	OLDER	M	72	81.8	30	8.1
7	OLDER	F	68	100.0	27	10.5
8	OLDER	M	74	100.0	29	6.1
9	OLDER	F	66	90.0	29	7.1
10	OLDER	F	69	100.0	29	9.1
11	OLDER	F	68	100.0	30	8.3
12	OLDER	F	73	100.0	30	8.1
13	OLDER	M	80	100.0	30	9.5
14	OLDER	F	68	100.0	29	11.4
15	OLDER	M	77	80.0	27	9.1
16	YOUNG	F	25	86.7	30	5.6
17	YOUNG	M	23	100.0	29	7.0
18	YOUNG	F	22	88.9	30	7.0
19	YOUNG	F	26	66.7	30	5.2
20	YOUNG	F	25	100.0	30	11.0
21	YOUNG	M	28	79.0	30	8.1
22	YOUNG	M	22	94.1	30	7.3
23	YOUNG	M	24	95.0	30	6.9
24	YOUNG	M	22	100.0	30	10.2
25	YOUNG	M	20	84.6	30	5.1
26	YOUNG	F	24	100.0	30	8.5
27	YOUNG	F	22	89.5	30	8.6
28	YOUNG	M	20	86.7	29	10.8
29	YOUNG	F	25	100.0	29	8.2
30	YOUNG	F	23	100.0	30	10.5

**Table 2:** Tissue fractions and quality metrics (mean ± SD) of the MRS data extacted using the GANNET toolbox are shown for young and older adults. P-values in bold indicate a significant difference between groups. Group differences in gray matter, white matter, cerebrospinal fluid and fit error were tested using the independent samples *t*-test. For frequency drift the Wilcoxon / Kruskal-Wallis Test was applied because while the distribution of frequency drift values was normal for young adults, it was non-normal for older adults..

Sensorimotor voxel			
**Tissue fraction**	Young	Older	*p* value
Gray matter	0.36 ± 0.04	0.28 ± 0.03	**<0.001**
White matter	0.56 ± 0.04	0.57 ± 0.05	0.739
Cerebrospinal fluid	0.08 ± 0.02	0.15 ± 0.04	**<0.001**
**Quality metric**			
Frequency drift	0.79 ± 0.39	0.68 ± 0.33	0.395
Fit error	6.07 ± 1.49	6.28 ± 2.23	0.763
